# Statistical Permutation Test Reveals Progressive and Region-Specific Iron Accumulation in the Thalami of Children with Aspartylglucosaminuria

**DOI:** 10.3390/brainsci10100677

**Published:** 2020-09-27

**Authors:** Viljami Sairanen, Anna Tokola, Ritva Tikkanen, Minna Laine, Taina Autti

**Affiliations:** 1 HUS Medical Imaging Center, Radiology, University of Helsinki and Helsinki University Hospital, 00029 Helsinki, Finland; anna.tokola@hus.fi (A.T.); taina.autti@hus.fi (T.A.); 2 Department of Computer Science, University of Verona, 37134 Verona, Italy; 3 Institute of Biochemistry, Medical Faculty, University of Giessen, Friedrichstrasse 24, 35392 Giessen, Germany; ritva.tikkanen@biochemie.med.uni-giessen.de; 4 Helsinki University Central Hospital, Child Neurology, 00029 Helsinki, Finland; minna.laine@hus.fi

**Keywords:** aspartylglucosaminuria, aspartylglucosaminidase, magnetic resonance imaging, susceptibility-weighted imaging, medical image analysis, gene therapy, pharmacological chaperone mediated therapy

## Abstract

Aspartylglucosaminuria (AGU) is a rare lysosomal storage disorder causing developmental delay, intellectual disability, and eventual death. A distinct feature in AGU is iron accumulation within the thalamus. Our aim is to demonstrate that susceptibility-weighted images (SWI) could be used as an MRI biomarker to evaluate the response within the AGU population to newly evolving treatments. SWI from 16 patients with AGU and 16 age-matched controls were used in the analysis. Thalamic volume with an iron accumulation was identified using a permutation test. Group differences were investigated for both the complete thalamus and the iron accumulation regions. Group-wise age correlation within these volumes were assessed with analysis of variance and multivariate regression. We found a statistically significant and large difference (*p*-value = 0.01, Cohen’s D = 0.97) for the whole thalamus comparison and an even greater difference in the iron accumulation regions (*p*-value < 0.01, Cohen’s D = 3.52). Furthermore, we found strong evidence for iron accumulation as a linear function of age with R^2^ = 0.65 only for AGU. The statistical analysis of SWI provides tools for assessing the degree of iron accumulation. This method could be used to study the response to treatments, in that a successful treatment would be expected to result in a decline in iron accumulation.

## 1. Introduction

The rare lysosomal storage disorder aspartylglucosaminuria (AGU) is known to inflict a continuous deterioration in the cognitive and motor skills of the patients. AGU is a generalized disease affecting the whole body, including the brain. AGU is usually diagnosed by demonstrating the accumulation of glycoasparagines in the urine of the patients or by genetic diagnosis. In specific cases, the activity of the aspartylglucosaminidase enzyme can also be measured in patient serum or cells. For a more detailed elaboration of AGU as a disease, we would like to recommend two previous publications [1,2]. In short, children with AGU appear healthy as newborns. The first symptoms noted in early childhood include abnormally slow development of motor skills and speech. A gradual decline in cognitive and motor skills starts in childhood and speeds up after the age of 25 to 28 years, leading to severe intellectual disability, with death typically before the age of 50 years [3]. The neuropathological features of AGU include myelination defects that can progress to demyelination with age. In addition, some degree of brain atrophy can also be also observed in older AGU patients [1].

Currently, there is no approved treatment available to cure or even to slow down the progress of AGU, even though the disease is known to result from a mutation in the *AGA* gene [4]. Recently, small molecule compounds were identified as possibly suitable for pharmacological chaperone-mediated therapy for patients with AGU [5], and a clinical treatment trial was initiated. Furthermore, several preclinical studies aiming at enzyme replacement or gene therapy or immunomodulatory therapy in AGU have been published [6,7,8]. Thus, in the near future, there will be need for reliable methods to assess the response of patients to these treatments.

Susceptibility weighted imaging (SWI) is an MRI technique sensitive to para and diamagnetic materials, and it thus can be used, e.g., to detect iron accumulation in tissues. The accumulation of excess iron has been previously linked to lysosomal diseases [9], including AGU [1]. Especially, studies on Alzheimer’s disease and multiple sclerosis have utilized SWI’s sensitivity to iron [10,11,12,13,14]. As SWI is sensitive towards substances with an intrinsic property of magnetic susceptibility, substrates such as proteins that do not contain iron but might also accumulate in the brain are virtually invisible for the technique. Based on the previous visual findings [1], we hypothesize that (i) iron accumulates in specific regions of the thalamic nuclei, and (ii) as the AGU disease progresses with age, the iron accumulation becomes more pronounced.

In this study, we present a statistical segmentation method of the thalamic nuclei into specific iron-accumulation regions that are typical in AGU. Furthermore, we propose an analysis framework for evaluating the degree of iron accumulation in these regions and demonstrate the differences in two populations hereafter referred to as “patients with AGU” and “controls”. The AGU population is a group of patients with AGU disease, whereas the controls are age-matched healthy individuals. Combination of the proposed methods could serve as an MRI-based biomarker to study both the progression of a disease with iron accumulation and the effectivity of any treatment in patients with such diseases.

## 2. Materials and Methods

This study was approved by the ethical reviewing board for Women, Children, and Psychiatry of the Helsinki ja Uusimaa region (License number HUS/2604/2017, granted 19 October 2017). A written informed consent for the study and for data handling was obtained from the parents of the probands. All patients included in this study are homozygous for the major Finnish AGU variant AGU_Fin-major_. The patient cohort has been described more in detail by Tokola et al. [1].

### 2.1. MRI Acquisition

In this study, we analyzed data from 16 patients with AGU (8 female and 8 male, 3.4–17.0 years of age, with a mean of 10.3 ± 4.0 years) and 16 age-matched controls (10 female and 6 male, 7.1–16.7 years of age, with a mean of 11.6 ± 3.2 years) acquired using a 3T MAGNETOM Skyra (Siemens, Erlangen, Germany) scanner. The MRI protocol included an SWI sequence with TR of 27 ms, TE of 20 ms, flip angle of 15°, slice thickness of 2.0 mm, and in-plane resolution 0.86 mm × 0.86 mm with a matrix of 256 × 232. The scanner manufacturer uses a so-called “left-handed” reference scheme in the SWI filtered-phase images, which results in an increase in the measured phase-filtered SWI signal. The patients are participating in an interventional clinical trial, and they were imaged before the onset of the treatment [1].

### 2.2. Image Registration and Segmentation of Thalamic Volume

**Figure 1:**
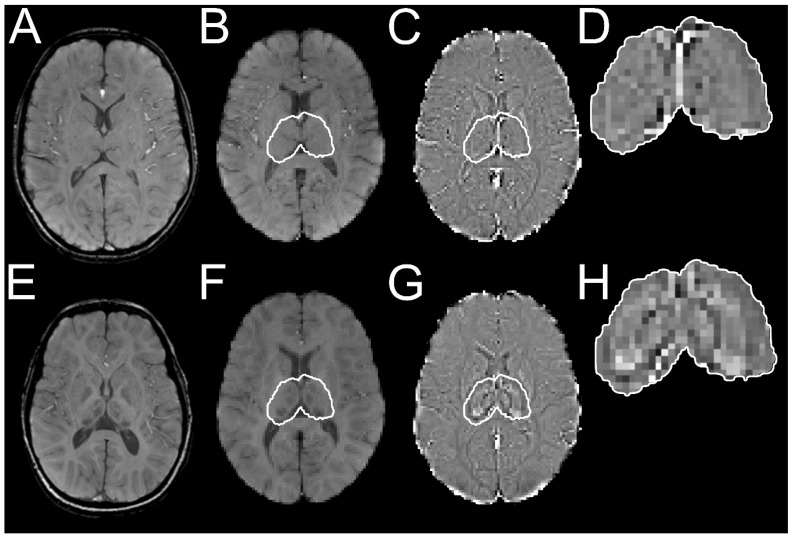
Visualization of the segmentation pipeline of the whole thalamus. An axial slice from a control is shown on the upper row. (**A**) Magnitude susceptibility-weighted images (SWI) are registered to MN152 space shown in (**B**) where the thalamus is highlighted with a white contour. (**C**) The obtained transformations are applied to phase-filtered SWI for the segmentation shown in (**D**), from which the average and voxel-wise thalamic intensities are derived. Respective steps for a patient with aspartylglucosaminuria (AGU) depict iron accumulation in (**E**–**H**). Iron is seen as an intensity decrease in magnitude images (**E**,**F**) or an intensity increase in the phase-filtered images (**G**,**H**).

All magnitude SWI were registered to MNI152 T1-weighted image with 1.0 mm isotropic resolution using Advanced Normalization Tools version 2.1.0 [15]. The accuracy of image transformations for every subject was confirmed by visual analysis. Thalamic regions were segmented by applying the same transformations on the phase-filtered SWI, which were then segmented using the thalamus region of the MNI152 structural atlas. This segmentation process is visualized in Figure 1 for one control, and one patient with AGU, of similar ages.

### 2.3. Permutation Test-Based Location of the Iron Accumulation Regions

We developed a statistical voxel-wise permutation test to locate the iron accumulation regions in thalamus. Our null-hypothesis was that the patients with AGU do not have a higher signal intensity in the phase-filtered SWI due to iron accumulation, with the alternative hypothesis that patients with AGU exhibit a higher signal intensity than the controls. This hypothesis is based on the prior knowledge that higher iron concentrations in the tissue result in a brighter signal in phase-filtered SWI in a ‘’left-handed’’ reference system [16]. This hypothesis was also confirmed by the statistical analysis explained in the Section 2.4.

We defined the difference between the groups in the permutation test as an absolute value of the remainder of the average voxel-wise intensity within each group. The permutation test was non-exact, with 10,000 permutations, as the number of permutations for an exact test would be approximately 6.3 × 10^21^ with the 16 subjects in each group. Subject-wise age and sex were used as covariates. To increase the power of the permutation test, the statistical images for each permutation were enhanced using a threshold-free clustering [17]. Finally, the Bonferroni correction was applied to account for family-wise errors due to multiple comparisons over all the voxels within the thalamus.

### 2.4. Statistical Analysis

The analysis was performed using an average of phase-filtered SWI signal intensity measured from (i) the whole thalamus and (ii) from the iron accumulation regions. The statistical analysis pipeline was divided into two parts: the first part consisted of group comparison (steps 1 to 3), and the second part of age-related effects (steps 4 to 6). In step (1), group-wise boxplot was investigated to identify differences between the group means and variances. In step (2), the effect size was measured using Cohen’s D for unequal variances. In step (3), two-sample t-test with unequal variances was performed to identify if the difference between group means was significant. In step (4), a two-way analysis of variance was used to test for differences in the expected intensities between the groups and the continuous factor age. In step (5), a multivariate linear regression analysis was used to study the age dependency of the average intensity of both groups. In step (6), analysis of covariance was done to evaluate if the difference was due to the age term or the interaction term of Group*Age, which models the degree of iron accumulation and corresponds to the slope calculated in the step 5.

## 3. Results

### 3.1. Segmentation and Permutation Test to the Locate Iron Accumulation Regions

**Figure 2:**
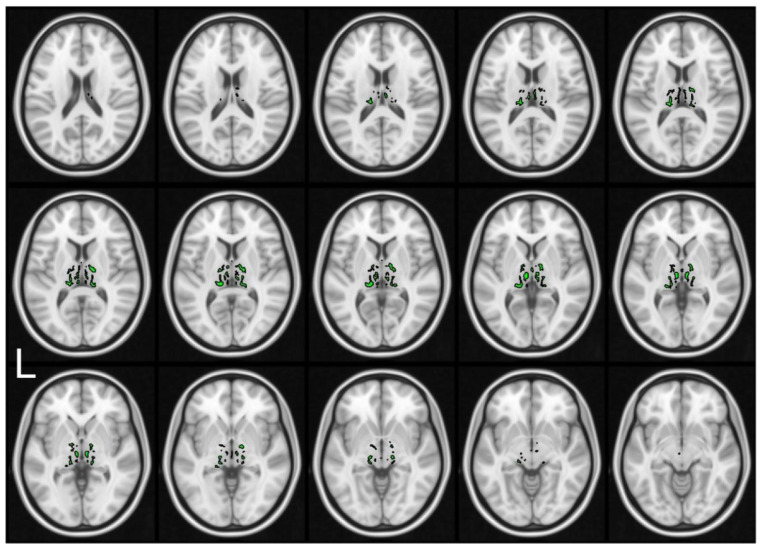
Iron accumulation regions are shown as contoured green overlaid on the brain T1-weighted MNI152 atlas image. Regions were located within the thalami of patients with AGU using a voxel-wise permutation test, demonstrating that the iron accumulation is not uniform within thalamus but concentrates in specific clusters.

SWI images were registered as described above, and the thalamic regions were identified as described previously [1]. We focused our analysis on the thalami, since our previous findings have shown that there is a profound iron accumulation in this region in AGU. Furthermore, we did not intend to analyze the whole brain, as we aimed at developing and testing a statistical method that could be used for the measurement of iron accumulation in specific, restricted brain regions. However, it is possible that further regions of the brain of AGU patients also exhibit iron accumulation. Thalami were segmented into the iron accumulation regions based on the statistically significant findings with *p*-value < 0.05 in the permutation test that were corrected for the family-wise error. Iron accumulation regions are visualized in Figure 2, and they indicate that the distribution of iron in the thalami of the patients with AGU is not uniform but specific to certain structures.

### 3.2. Statistical Analysis

In this section, we report results from the statistical analysis, which details are described in the Materials and Methods Section 2.4. Using the statistical analysis of the whole thalamus, we found modest differences in the group-wise means and variances, with no age-related correlation. The analysis of group-wise thalamic intensities (Step 1) resulted in a mean thalamic intensity of −57 for the control group and −41 for the patients with AGU. The corresponding variances were 342 and 242, favoring the use of unequal variances in the further analysis. The effect size (Step 2) was measured using Cohen’s D and was 0.97, demonstrating a large difference between the groups. The difference was deemed statistically significant (Step 3), as the *p*-value was 0.01 in the two-sample *t*-test.

**Figure 3:**
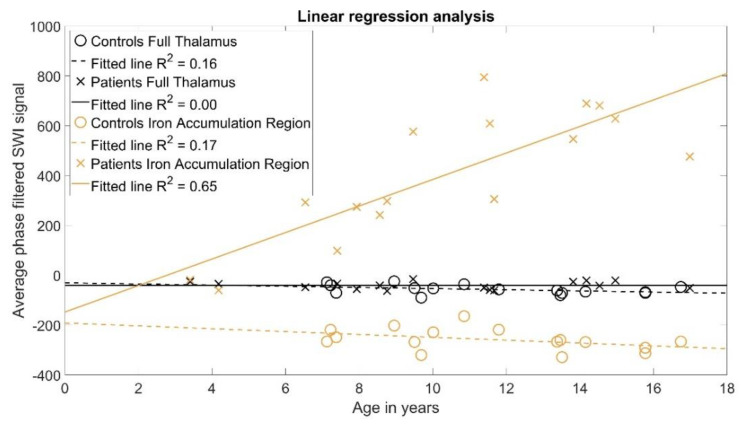
The multivariate linear regression analysis of the average intensities calculated from the whole thalamus (black) and the iron accumulation regions (orange). A strong linear correlation with R^2^ = 0.65 between age and phase-filtered SWI intensity, i.e., iron accumulation, was observed only in the iron accumulation region for the patients with AGU. Other tests did not indicate statistically significant linear correlations.

The statistical analysis of correlation between age and average intensities calculated from the whole thalamus using two-way analysis of variance (Step 4) indicated that there was insufficient evidence for an age-related effect as the *p*-value was 0.29. The multivariate linear regression coefficients (Step 5) are reported in Table 1A and Figure 3. While a significant difference in the intercept term was found, with a *p*-value < 0.01, there was no indication of significant linear correlations, i.e., differences in the slope term. Furthermore, linear fittings visualized in Figure 3 demonstrate that the lines for both groups are relatively horizontal with R^2^ of 0.16 for the controls and R^2^ of 0.0 for the patients with AGU. The analysis of covariance reported in Table 1B (Step 6) supports these findings, as the only significant term was the group difference with a *p*-value of 0.02.

In the statistical analysis of the iron accumulation regions of the thalamus, we found a large difference in the group-wise means and variances, with the intensities of the patients with AGU depicting a strong linear correlation with age. The analysis of group-wise thalamic intensities (Step 1) resulted in a mean thalamic intensity of −258 for the control group and 402 for the patients with AGU. The corresponding variances were 1952 and 68,216, favoring the use of unequal variances in the further analysis. The effect size (Step 2) was measured using Cohen’s D and was 3.52, demonstrating an extremely large difference between the groups. The difference was deemed statistically significant (Step 3), as the *p*-value was < 0.01 in the two-sample t-test.

The statistical analysis of correlation between age and average intensities calculated for the iron accumulation regions of the thalamus using two-way analysis of variance (Step 4) indicated that there was evidence for an age-related effect, as the *p*-value was < 0.01. The multivariate linear regression coefficients (Step 5) are reported in Table 2A and Figure 3. While a significant difference in the intercept term was found, with a *p*-value of 0.02, there was also indication of significant linear correlations, with the *p*-value for the slope term <0.01, suggesting that iron accumulates in patients with AGU to a much higher degree than in the controls. Furthermore, linear fittings visualized in Figure 3 show that the line for the controls is relatively horizontal, with R^2^ of 0.17, whereas the patients with AGU have a strong positive linear correlation with R^2^ of 0.65. The analysis of covariance reported in Table 2B (Step 6) supports these findings, as it demonstrates the significant differences between the group means with a *p*-value < 0.01 as well as a significant linear correlation with the slope interaction term Group × Age with a *p*-value < 0.01.

## 4. Discussion

In this study, we aimed at providing a method for the evaluation the iron accumulation in the thalamic nucleus of patients with AGU [2] using an MRI-based SWI technique that is sensitive towards susceptibility changes in tissues due to, e.g., excess iron [10,11,16]. The iron accumulation in AGU patients was first observed by visual inspection of the SWI acquired in a previous study [1]. Our current approach builds on this visual finding. We successfully used a statistical permutation test to confirm that iron does not accumulate throughout the whole thalamus, but rather in specific regions within it (Figure 2). Moreover, we proved that the iron accumulates in these regions to a significantly higher degree in the patients with AGU than in the controls (Figure 3). The proposed analysis method could serve as an MRI-based biomarker for both studying the progression of the AGU disease and, more importantly, assessing the effectivity of any emerging treatment for this disease.

Our main hypotheses were that (i) iron accumulates in specific regions in the thalamus, and (ii) the amount of iron in the thalamus accumulates as the patient with AGU grows older. These hypotheses were based on visual findings in a previous study [1]. The statistical tests used for the SWI agreed well with the visual findings, thus supporting our hypotheses. While we observed a large and statistically significant difference with Cohen’s D of 0.97 and a *p*-value 0.01 between the group-wise means calculated from the whole thalamus region in phase-filtered SWI, we were not able to detect any age-related correlation in these regions. However, after we further segmented the thalamus into iron accumulation regions using the permutation test, the differences within these regions became very pronounced and statistically significant, with Cohen’s D of 3.52 and *p*-value < 0.01. Furthermore, the patients with AGU had a strong linear correlation with R^2^ 0.65 between the phase-filtered SWI signal and their age. Analysis of covariance confirmed that this linear correlation was significantly different from the control group, with a *p*-value < 0.01, demonstrating that the degree of iron accumulation in the thalamus of a patient with AGU is indeed abnormal. Investigation of this slope term after a treatment in the patients with AGU could be used as an early and non-invasive indicator for the efficacy of the treatment.

We opted for a permutation test to locate the iron accumulation regions to avoid prior assumptions about the thalamic structures that could suffer from the iron accumulation. While use of thalamic atlases [18,19] could provide a better understanding of the underlying thalamic connectivity and functionality, they could also hide or mask parts of the located iron accumulation regions due to averaging over a larger volume instead of a voxel-based analysis.

The statistical analysis of the iron accumulation regions detected here, and of the whole thalamus, were performed separately to avoid comparing inter-dependent measurements. However, the results of multivariate linear regression were summarized in a single plot in Figure 3 to facilitate the comparison between the analyses of different volumes. In the future, a similar multivariate linear regression analysis could be performed on patients with AGU before and after treatment to observe if the slope term decreases. With an analysis of covariance between the pre- and post-treatment slopes, it would be possible to identify if the change in the slope is statistically significant, which would suggest that the treatment is effectively decreasing the iron accumulation.

Iron accumulation appears to be a general feature in many neurodegenerative diseases, as it has been observed in further lysosomal storage disorders, multiple sclerosis, and Alzheimer’s disease [9,10,11,12,13]. Therefore, SWI-based imaging to detect iron load may be a useful biomarker for clinical studies addressing the treatment of such diseases. As the duration of clinical SWI sequence is short, with less than 5 min of acquisition time, it might be especially suitable as an MRI-based biomarker for investigating children and adolescents with lysosomal diseases who tend to show hyperactivity and require either a fast acquisition or anesthesia.

Biomarkers that address the brain pathology are especially desirable in monitoring treatments that are peripherally administrated, since it is important to ensure that the treatment also improves the symptoms in the brain. However, such biomarkers are also useful in the case of direct central-nervous-system-targeted treatments such as gene therapy and enzyme replacement therapy [4,6,7,8], as they provide a non-invasive and quantitative means of monitoring the treatment effect.

### Limitations of This Study

The proposed image processing and registration pipeline could be improved by using a study-specific population average template instead of the MNI152 atlas, as the latter might not translate with the best accuracy to the brain images of very young children [15]. However, the study-specific average template would require manual segmentation of the thalamus and would still contain registration errors to some degree. The main benefit of this approach would be a more restricted area in which the thalamic iron accumulation would be studied using permutation tests. The smaller volume would translate into smaller number of measurements, i.e., voxels, and thus reduce the need for multiple comparison corrections.

In our approach, we assumed that the average signal intensity represents the distribution of the measured phase-filtered SWI intensities. While averages over many measurements tend to follow a normal distribution due to the central limit theorem, it might not be the case with our small sample size. The permutation test could be repeated using Kolmogorov–Smirnov statistics or other similar statistics that describe the properties of the studied distributions with fewer assumptions. However, the difference between the patients with AGU and the controls was very large (Cohen’s D of 3.52), which indicates that the mean is an adequate choice to summarize the distributions.

## 5. Conclusions

The main finding of the present manuscript, as compared to the study by Tokola et al. [1], is that in the present study, we have developed a statistical segmentation method that makes it possible to quantitate the accumulation of iron in the brain of AGU patients at different stages of the disease. We show that in older patients, iron load increases, and it very strongly correlates with the age and progression of the disease. No iron accumulation was observed in our age-matched, healthy control probands. The correlation of iron with age in the patients was not demonstrated in our previous study [1] that described the potential regions of iron accumulation in the brains of AGU patients using the same cohort of patients as the present study. Therefore, the main merit of the present paper is the methodological development of the quantitative analysis of iron accumulation. With the present method, it will be possible in the future to longitudinally follow the degree of iron accumulation in the patients upon disease progression, and the method can thus be used for the diagnosis of AGU. Furthermore, our method will provide a valuable biomarker for clinical studies as it directly and non-invasively addresses the brain pathology. SWI is a widely used sequence in pediatric neuroimaging, and it is included in various clinical routine protocols, including the suspicion of metabolic disorders in pediatric patients. Therefore, evaluating the iron accumulation with this novel method using clinical SWI phase images would provide an alternative for QSM (quantitative susceptibility mapping). Very importantly, the method developed in the present study may also be suitable for further (lysosomal) disorders that show a similar iron accumulation.

## Figures and Tables

**Table 1:** The whole thalamus test results between age and average image intensity.

**(A). Multivariate regression coefficients f(x) = *A* × x + *b***					
		**Estimate**	**Std. Err.**	**T**	***p*-Value**
Intercept *b*		−35.38	10.15	−3.49	<0.01
Δ*b* Controls		+4.86	10.15	+0.48	0.64
Δ*b* Patients with AGU		−4.86	10.15	−0.48	0.64
Slope *a*		−1.17	0.87	−1.34	0.19
Δ*a* Controls		−1.14	0.87	−1.30	0.20
Δ*a* Patients with AGU		+1.14	0.87	+1.30	0.20
**(B).** **Analysis of Covariance**					
	**df**	**Sum. sq.**	**Mean sq.**	**F**	***p*-Value**
Group	1	1861.50	1861.50	6.56	0.02
Age	1	338.20	338.20	1.19	0.28
Group × Age	1	480.74	480.74	1.69	0.20
Error	28	7945.12	283.75		

**Table 2:** The iron accumulation region test results between age and average image intensity.

**A. Multivariate Regression Coefficients f(x) = *a* × x + *b***					
		**Estimate**	**Std. Err.**	**T**	***p*-Value**
Intercept *b*		−169.77	70.22	−2.42	0.02
Δ*b* Controls		−22.02	70.22	−0.31	0.76
Δ*b* Patients with AGU		+22.02	70.22	+0.31	0.76
Slope *a*		23.73	6.04	3.93	<0.01
Δ*a* Controls		−29.46	6.04	−4.87	<0.01
Δ*a* Patients with AGU		+29.46	6.04	+4.87	<0.01
**B.** **Analysis of Covariance**					
	**df**	**Sum. sq.**	**Mean sq.**	**F**	***p*-Value**
Group	1	3,771,650.55	3,771,650.55	277.69	<0.01
Age	1	349,463.43	349,463.43	25.73	<0.01
Group × Age	1	322,764.71	322,764.71	23.76	<0.01
Error	28	380,303.90	13,582.28		
